# Can Meditation Influence Quality of Life, Depression, and Disease Outcome in Multiple Sclerosis? Findings from a Large International Web-Based Study

**DOI:** 10.1155/2014/916519

**Published:** 2014-11-12

**Authors:** Adam B. Levin, Emily J. Hadgkiss, Tracey J. Weiland, Claudia H. Marck, Dania M. van der Meer, Naresh G. Pereira, George A. Jelinek

**Affiliations:** ^1^School of Medicine,The University of Melbourne, Melbourne, VIC 3010, Australia; ^2^Emergency Practice Innovation Centre, St. Vincent's Hospital, Melbourne, VIC 3065, Australia; ^3^Department of Epidemiology and Preventive Medicine, Monash University, Prahran, VIC 3800, Australia; ^4^Medical School, University of Notre Dame, Fremantle, WA 6959, Australia

## Abstract

*Objectives*. To explore the association between meditation and health related quality of life (HRQOL), depression, fatigue, disability level, relapse rates, and disease activity in a large international sample of people with multiple sclerosis (MS). *Methods*. Participants were invited to take part in an online survey and answer questions relating to HRQOL, depression, fatigue, disability, relapse rates, and their involvement in meditation practices. *Results*. Statistically and potentially clinically significant differences between those who meditated once a week or more and participants who never meditated were present for mean mental health composite (MHC) scores, cognitive function scale, and health perception scale. The MHC results remained statistically significant on multivariate regression modelling when covariates were accounted for. Physical health composite (PHC) scores were higher in those that meditated; however, the differences were probably not clinically significant. Among those who meditated, fewer screened positive for depression, but there was no relationship with fatigue or relapse rate. Those with worsened disability levels were more likely to meditate. *Discussion*. The study reveals a significant association between meditation, lower risk of depression, and improved HRQOL in people with MS.

## 1. Introduction

Multiple sclerosis (MS) is a common chronic neurological disease [1, 2] typified by neuronal demyelination and inflammation, leading to axonal injury with disease progression [2]. There are several categories of MS, with relapsing-remitting being the most common [3]. MS is currently incurable; however, there are many strategies used for disease management, including pharmacological agents, lifestyle modification, rehabilitation, psychosocial support, and rarely surgery, all of which have varying efficacy [4–6].

It has been proposed that stress may play a role in the MS disease course, given that it has been shown to have a strong association with frequency of disease relapses [7, 8]. It is thought, therefore, that strategies that reduce and manage stress may play a role in secondary or tertiary prevention for people with MS by slowing disease course and improving quality of life (QOL). One such strategy is meditation. Interest in meditation as a medical treatment was generated by Kabat-Zinn's research showing that meditation could be a useful tool for managing chronic pain [9]. Meditation, which encompasses a wide range of techniques, has been associated with symptom reduction in psychiatric conditions, electroencephalography (EEG) changes and beneficial structural brain changes on neuroimaging with long term use [10–18]. More specifically meditation has been associated with stress reduction, decreased rate of maladaptive coping strategies, and increased resilience for those with MS [19].

A randomised controlled trial by Grossman et al. is, to date, the strongest evidence for the efficacy of meditation on MS morbidity outcomes [20]. In the study 150 participants were randomised into a mindfulness based intervention program or a control group of standard treatment, with those in the experimental group having significantly improved health related quality of life (HRQOL) as well as decreased depression, anxiety, and fatigue up to six months after the intervention. Other interventional studies have also found clinically significant benefits of meditation on MS [21–24]; however these studies were not as methodologically rigorous as that led by Grossman.

Further evidence to support the use of meditation as a successful management adjunct for MS exists primarily in the form of cross-sectional studies. These studies also highlight a positive association between meditation and HRQOL in MS; however they are often limited by difficulty in verification of the MS diagnosis [25–27], being limited to one or two countries [19, 25, 27, 28], not employing validated instruments [26–28], and small sample size [19, 28].

While some evidence exists that meditation may have a beneficial effect on HRQOL for those with MS, none of the current literature has demonstrated any significant positive effect on decreasing relapse rates. This study is part of a research project “Health Outcomes and Lifestyle Interventions in a Sample of People with Multiple Sclerosis” (the HOLISM study). Previous studies published from the same dataset have examined the association between health related outcomes in MS, omega 3 supplementation and fish consumption [29], other dietary factors [30], and alcohol and smoking [31]. The aim of this study was to explore the relationship between meditation frequency and HRQOL, depression, fatigue, disability status, relapse rate, and disease activity in a large international sample of people with MS.

## 2. Methods

### 2.1. Participants and Recruitment

Participants were asked to complete an online survey on SurveyMonkey, reading a participation statement and giving consent prior to starting the survey. Participants aged 18 years or over who were diagnosed with MS by a physician were invited to take part. Ethics approval was granted by St. Vincent's Hospital Melbourne Human Research Ethics Committee (LRR 055/12). More detailed methodology has previously been described [32].

### 2.2. Data Collection and Tools

The study collected demographic details, including age, gender, education status, and current employment status, and diagnostic details, including type of MS, whether MS was diagnosed by a clinician, and years since diagnosis. All data were self-reported.

### 2.3. Health Related Quality of Life

The Multiple Sclerosis Quality of Life-54 survey (MSQOL-54) was used to measure HRQOL. This well validated tool comprises the 36-item short form health survey (SF-36) and 18 disease specific questions. 54 items give rise to 12 scales, 2 separate items, and a mental health composite (MHC) and a physical health composite (PHC) [29, 33–35]. Physical and mental health composites and subscales were calculated as per the scoring method from the MSQOL-54.

### 2.4. Disability

Disability was assessed using the Patient-Determined Disease Steps (PDDS), a tool that has been used by the North American Research Committee on Multiple Sclerosis registry (NARCOMS) as a surrogate for the Expanded Disability Status Scale (EDSS) [36–39]. The EDSS is commonly used to assess gait disability and correlates well with the PDDS which scores level of disability from 0 (normal) to 8 (bed bound). The PDDS was collapsed to form three ordinal categories: mild (scores 0–2), moderate (3–5), and major disability (6–8).

### 2.5. Relapse Rate

Participants were asked to recall how many medical doctor-diagnosed MS relapses they had in the previous 12 months and five years. A categorical variable “disease activity” was created by assessing whether 12-month relapse rate was higher, the same, or lower than participants' 5-year annualised relapse rates. For the purpose of analysis, only people with a relapsing-remitting type of MS were included for comparison of relapse rate or disease activity.

### 2.6. Depression

Depression risk was assessed using the two-item Patient Health Questionnaire-2 (PHQ-2), a shortened version of the PHQ-9, which has been validated in an MS population. This tool has a reported specificity of 92% and sensitivity of 83% for major depression with a cut-off score ≥ 3 [40].

### 2.7. Fatigue

Fatigue was assessed using the Fatigue Severity Scale (FSS), a tool which has been validated for use in an MS population [41, 42]. The FSS is a nine-question survey with answers being scored from 0 (least severe) to 7 (most severe). A total score above 4 was considered an indication of clinically significant fatigue, as determined in the literature [43].

### 2.8. Meditation

Participants were asked to provide an average weekly frequency of meditation in the last 12 months with responses including never, less than once per week, once to twice a week, three to four times a week, five to six times a week, and every day.

Meditation frequency variables were collapsed for meaningful analysis:

1. collapsed to two categories—never meditates or has meditated in the last 12 months;
2. collapsed to three categories—never meditates, meditates less than once per week, or meditates one or more times per week.

No specific definition of meditation was provided to participants who were simply asked how often and for how long they participated in meditation.

### 2.9. Data Analysis

Data were analysed using IBM SPSS version 21.0.

Only participants reporting that their diagnosis had been confirmed by a medical doctor were included in the analysis; those with clinically isolated syndrome or possible MS were excluded.

Categorical demographic data were analysed using cross tabulation to create contingency tables with Pearson's Chi Square or Fisher's Exact Test and adjusted standardised residuals (ASR) used to determine over- and underrepresentation of specific demographic groups participating in meditation. Results were only accepted if all cells had an expected cell count greater than 5 and results were considered significant if ASR were ≥ 2 or ≤−2 and alpha was < 0.05.

Bivariate analyses were undertaken to explore the relationship between meditation and each variable. When analysing 12-month relapse rates only those who had relapsing-remitting MS and had relapses confirmed by a physician were included.

T-tests were used to analyse significant differences in continuous variables between those that did and did not meditate (dichotomous variable), using Levene's test for assessment of equal variance.

Analysis of the categorical meditation data was undertaken using cross tabulation with categorical data (as described above) and one way ANOVA for comparison with continuous data. For all ANOVA, homogeneity of variance was assessed with Levene's test. Post hoc analyses were performed for significant group differences using Tukey HSD or Games Howell to assess paired differences, depending on homogeneity of variance.

Multivariate analysis was performed to determine independent predictor variables for outcome variables that were significant in bivariate analyses: MSQOL-54 composites, PDDS, and PHQ-2. Multiple regression (Enter method) was used for MSQOL-54 composites. Tests were performed to assess normality, linearity, and homoscedasticity and to check for outliers. Absence of multicollinearity was assessed with variance inflation factor (<4) and correlations (<0.7). Binary logistic regression was used for PHQ-2 with goodness of fit being assessed with omnibus and Hosmer and Lemeshow test. Ordinal logistic regression was used to assess PDDS. Assumptions were tested using: proportional odds assumption (test of parallel lines > 0.05), goodness of fit, and multicollinearity as above. Significance was assumed if two-tailed tests were below 0.05.

Given the large sample size there were enough participants to satisfy the requirements for all the tests used. Similarly, the central limit theorem was assumed for analyses involving continuous data.

## 3. Results

### 3.1. Demographics

There were 3132 respondents to the HOLISM study overall, and 2469 fit the inclusion criteria of being 18 years or over and having physician diagnosed MS. Of these 82.3% were female. The median age was 46 years (IQR = 30.5–60.5) and median age at diagnosis 37 years (IQR = 22–52). Almost two-thirds of respondents had relapsing-remitting MS. Respondents were largely from the USA (32.7%), Australia (25.5%), and the UK (16.9%). Further demographic data from the HOLISM have been published previously [32].

**Figure 1:**
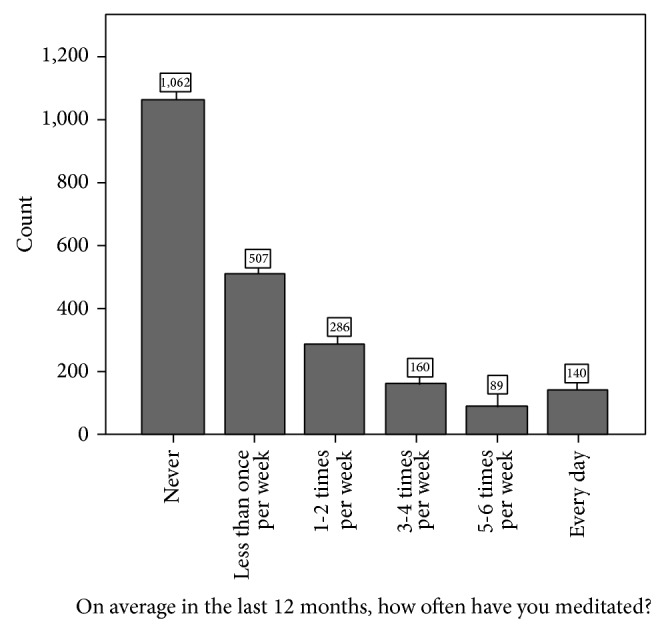
Meditation frequency of survey respondents.

Of the 2469 respondents, up to 2244 (90.9%) completed the meditation section of the HOLISM survey. 1182 (52.7%) reported having meditated at least once in the past twelve months (Figure 1).

Women, those with bachelor degrees or higher, those working part time, and those in the age category 50–59 years were all more likely to have participated in meditation in the past twelve months (Table 1). There was no statistically significant association between meditation status and years since diagnosis of MS (data not shown).

### 3.2. HRQOL

PHC and MHC scores were significantly higher in respondents who meditated compared to those who did not (*P* = 0.001 and <0.001, resp.). Similarly the overall quality of life, energy, health distress, emotional well-being, health perception, and cognitive function HRQOL scales were all significantly higher in those who meditated (*P* < 0.001). Sexual health, pain, and social function HRQOL subscore mean differences were not related to meditation (data not shown).

Analyses showed a significant difference in MHC and PHC for frequency of meditation. Post hoc comparisons (Table 2) showed participants who meditated once or more per week had a higher MHC mean score compared to those who never meditated and those who meditated less than once per week, both of which were statistically significant. There was no significant difference between those who meditated less than once a week and those who never meditated. Identical analysis of the PHC demonstrated a significantly higher mean PHC score for participants who meditated once or more per week compared to those who never meditated and for those who meditated less than once per week, when compared to those who never meditated. There was no significant difference between those who meditated less than once a week and those who meditated at least once per week (Table 2).

Multiple regression analysis was conducted to predict PHC and MHC from age, gender, education level, and frequency of meditation. The models generated accounted for 8.1% of PHC variance and 3.0% of MHC variance (Table 3).

After adjusting for age, gender, and education, participants who meditated once or more per week had a significantly higher mean MHC compared to those who never meditated, whereas there was no significant difference between participants who meditated less than once a week and those that never meditated (*P* < 0.001 and *P* = 0.513, resp.). The same adjustments for PHC found that the mean score was significantly higher for those who meditated once or more per week or less than once per week compared to those who never meditated (*P* = 0.009 and *P* = 0.031, resp.). However, there was no significant difference between the two categories of people that did meditate.

### 3.3. Depression Risk

Those who had meditated in the last year were less likely to screen positive for depression compared to those who had not (*P* < 0.001). A significant association was found also with frequency of meditation (*P* < 0.001) (data not shown).

Logistic regression controlling for gender, age, and level of education showed that compared to those who never meditated, those who meditated once or more per week had half the odds of screening positive for depression (Table 3).

### 3.4. Disability

There was no association between those who did and did not meditate and PDDS score. However, there was a significant association between frequency of meditation and PDDS score (*P* < 0.001), with those who were more disabled meditating more frequently (data not shown).

Ordinal logistic regression was undertaken to model PDDS, controlling for gender, age, and level of education; the model predicted that the odds of increased disability for those who meditated less than once per week were 25% lower compared to those who never meditated (Table 3). Meditating once or more per week compared to not meditating did not predict disability.

### 3.5. Clinically Significant Fatigue

There was no significant relationship between those who did and did not meditate and FSS scores. Therefore regression analysis was not explored.

### 3.6. Relapse Rate

For the subset of people with relapsing-remitting MS, there was no effect of ever meditating or frequency of meditation on the mean number of 12-month relapses. Therefore, regression analysis was not used to further explore relapse rate and meditation.

### 3.7. Disease Activity

Among people with relapsing-remitting MS, there was no significant association between whether disease activity was decreasing (improving), stable or increasing (worsening), and meditation status or frequency of meditation. Given the bivariate results were insignificant no regression analysis was performed.

## 4. Discussion

This study is the first cross-sectional survey to focus solely on the association between meditation and MS health related outcomes. Well-educated women aged 50–59 with relapsing-remitting MS were the most commonly represented demographic in this survey. This is consistent with relapsing-remitting MS being the most common form of the disease [3] and MS being a disease which more commonly affects females (although our sample had an even higher proportion of females than usually seen in MS cohorts) with a typical onset between 20 and 40 years of age [44]. Participants in the study were mostly English speaking from Western countries; however, there was a more culturally and geographically diverse population than in previous similar studies [25, 27].

In our study, those most likely to meditate were women, those over 50 years of age, participants who worked part time, and those with bachelor degrees or higher. Just over 50% of participants reported having meditated at least once in the last 12 months and 30% reported meditating at least weekly. The rate of meditation in this study is higher than that found in previous papers reporting meditation rates in MS to range from 9 to 25% [25–28, 45]. This may be due to the HOLISM study recruiting participants from online platforms, including sites which promoted a holistic management approach to MS, resulting in a strong representation of people who were interested in self-management of their condition.

Our study provides support for work done by Grossman et al. [20]; using different methodology in a much larger, geographically diverse sample, we have shown similar associations between meditation and a variety of quality of life outcomes. Meditation was significantly associated with higher mean scores in both the mental and physical health composite as well as in many of the 12 MSQOL-54 scales. HRQOL measurements are derived from the SF-36 and it is generally accepted that an improvement in this scale of five points is clinically significant [46, 47]. As such, a clinically significant benefit in MHC may exist for those who meditated once a week or more compared to those who never meditated. This association held in multivariate analysis controlling for age, gender, and education level.

Meditation was statistically and possibly clinically significantly related to higher health perception and cognitive function subscores. Much of meditation is involved with observing thoughts and emotions and exploring them without becoming reactive to them [12, 16, 17]. These results suggest that being able to explore emotions may assist people with MS to have a better understanding and acceptance of the disease and therefore improved perception of health status. Furthermore, a more relaxed state of mind and increased levels of concentration associated with meditation [19] may allow for improved cognitive function in those with MS who meditate.

Physical health composite scores were found to be significantly higher in meditators on bivariate and multivariate analysis; however the improvement did not satisfy the clinical significance criteria above. As such, more thorough analysis of the relationship between the PHC and meditation status is warranted in future studies.

Similarly, a statistically significant increase was seen in almost all the other MSQOL-54 scales; however other than those above they may not have been clinically significant. The varying efficacy of meditation on different scales and composites of the MSQOL-54 suggests that the relationship between HRQOL and meditation status is a complex one.

Importantly, participants who meditated had markedly lower depression risk. Multivariate analysis supported a significant relationship between frequency of meditation and decreased risk of depression for those who meditated once or more per week compared to those who never meditated. While the results were suggestive, more in depth analysis into the finding may be necessary given that the significance of the effect was lost when participants who meditated less than once a week were compared to participants who never meditated.

Interestingly, those who meditated once or more per week were significantly more likely to have a higher level of disability, as assessed using the PDDS. This finding may suggest that those with greater disability are more likely to meditate, perhaps feeling a greater need to meditate as a result of their condition. Our analyses showed that those aged over 50 years were more likely to meditate, so it may be that many people with MS begin to consider meditation as an adjunct to treatment as they reach middle age and this may coincide with the natural timing of disease progression in MS, a possibility given that age was shown to be a significant variable on regression analysis.

Results for fatigue, relapse rate, and disease activity were less robust. Results for each of these categories were statistically insignificant, with no association demonstrated between relapse rates and disease activity with frequency of meditation. Given that meditation appears to be associated with depression, it is surprising that a similar relationship was not observed with fatigue, as the literature indicates that these two conditions are commonly intertwined [48, 49].

A deeper appreciation of the biological association between meditation and MS disease course is needed, given the mixed results demonstrated in our analysis. A better understanding of the biological relationship will enhance our understanding of meditation as a preventive strategy for those with MS. Cortisol, a hormone secreted by the anterior pituitary in response to stress, may be involved in the mechanism by which practising meditation is able to alter MS disease course. Previous studies have shown a decrease in cortisol after sustained meditation [50, 51]. Such a decrease in cortisol, which is often elevated in people with chronic conditions such as MS [52], may contribute to improved HRQOL for those who practise meditation. Meditation has also been shown to alter brain structure and function [10–18]. While the mechanisms for these changes remain unknown, this too could be behind the improvement in HRQOL seen in those with MS who practise meditation. Although the exact mechanism remains unknown, this study showed that meditation may be associated with a clinically significant improvement in HRQOL and depression for those with MS. Given these positive findings, further investigation into the relationship between meditation and MS is warranted.

### 4.1. Limitations

Our data may have been susceptible to recall difficulties given that responses were self-reported and participants were asked questions regarding their disease and habits over long time periods. This would be especially true of recalling number of relapses in the last 12 months and five years as well as frequency of meditation in the last 12 months. Furthermore participants were required to self-report physician diagnosis of MS, a factor which could not be verified clinically, given the size and geographic diversity of the cohort.

There may have been responder bias given that there were no compulsory questions in the HOLISM survey. As a result respondents who had experience with meditation may have been more likely to answer the questions relating to meditation, although the item response rate is high at over 80%. Furthermore, although data were deidentified after collection, participation was not anonymous which could also have affected participants' tendency to over- or understate their responses based on what they believed the researchers were wanting to achieve. There may have been further response bias as a result of the survey being internet based, meaning that respondents were required to have access to the internet and be somewhat computer literate.

Meditation encompasses a wide range of nonhomogenous activities. It is possible that some forms of meditation are more or less effective than others. Given this study did not discriminate between types of meditation, that is, no definition of meditation was given to participants who were simply asked whether or not they meditated, it is possible the results are not generalisable to all forms of meditation.

Reverse causation cannot be excluded. That is, those with an increased HRQOL choose to meditate rather than meditation being a contributing factor in their improved HRQOL. Similarly it could be, although it seems implausible, that meditation leads to greater disability rather than those with a greater disability being more likely to engage in meditation.

## 5. Conclusion

This study reveals a significant association between meditation and better HRQOL and lower risk of depression for those with MS. This further supports the role of lifestyle modification as secondary or tertiary preventive management in MS and highlights the need for further research into the association between meditation and health outcomes in MS. Finally, given our findings and those of others, clinicians may consider encouraging patients with MS to consider meditation as a management strategy.

## Figures and Tables

**Table 1:** Summary of demographics comparing those who meditated in the last 12 months to those who did not.

	Meditates			
	Yes, *n* (%)	No, *n* (%)	Total (%)	*P*
Gender				
Female	**981 (53.5)** ^*^	852 (46.5)^†^	1833 (100)	0.004
Male	176 (45.5)^†^	**211 (54.5)** ^*^	387 (100)	
Age				
18–29 years old	50 (44.2)	63 (55.8)	113 (100)	0.042
30–39 years old	291 (52.1)	268 (47.9)	559 (100)	
40–49 years old	348 (49.1)	361 (50.9)	709 (100)	
50–59 years old	**322 (55.7)** ^*^	256 (44.3)^†^	578 (100)	
60+ years old	127 (56.2)	99 (43.8)	226 (100)	
Education				
Did not complete school	17 (37)^†^	**29 (63)** ^*^	46 (100)	<0.001
Completed school/trade	376 (44.3)^†^	**472 (55.7)** ^*^	848 (100)	
Bachelor degree or higher	**775 (57.8)** ^*^	566 (42.2)^†^	1341 (100)	
Employment				
Work full time	353 (48.2)^†^	**380 (51.8)** ^*^	733 (100)	0.013
Work part time	**285 (59.5)** ^*^	194 (40.5)^†^	479 (100)	
Stay-at-home parent/carer	92 (53.8)	79 (46.2)	171 (100)	
Unemployed	88 (52.4)	80 (47.6)	168 (100)	
Retired due to disability	267 (51.9)	247 (48.1)	514 (100)	
Retired due to age	37 (52.1)	34 (47.9)	71 (100)	
Other (incl. students)	49 (47.6)	54 (52.4)	103 (100)	

^*^denotes significantly overrepresented as determined by standardised adjusted residuals.

^†^denotes significantly underrepresented as determined by standardised adjusted residuals.

**Table 2:** Mean difference in MSQOL54 scales and composite between meditation frequency categories.

	Meditation frequency			Mean difference *P*		
	Never	Less than once per week	Once or more per week	Never versus less than once per week	Never versus once or more per week	Less than once per week versus once or more per week
MSQOL-54 composites						
Mean MHC (95% CI)	65.1 (63.8–66.4)	66.4 (64.5–68.2)	70.7 (69.1–72.2)	*P* = 0.534	**P** < 0.001^*^	**P** = 0.001^*^
Mean PHC (95% CI)	57.6 (56.2–59.0)	61.0 (59.0–63.0)	60.8 (59.0–62.6)	**P** = 0.021^*^	**P** = 0.016^*^	*P* = 0.99
MSQOL-54 scales						
Overall QOL (95% CI)	65.4 (64.2–66.6)	67.1 (65.4–68.8)	69.9 (68.5–71.2)	*P* = 0.233	**P** < 0.001^*^	**P** = 0.035^*^
Emotional well-being (95% CI)	67.3 (66.2–68.5)	67.1 (65.6–68.7)	72.7 (71.4–74.0)	*P* = 0.991	**P** < 0.001^*^	**P** < 0.001^*^
Energy (95% CI)	41.3 (39.9–42.6)	43.5 (41.6–45.5)	47.5 (45.8–49.2)	*P* = 0.140	**P** < 0.001^*^	**P** = 0.008^*^
Health perception (95% CI)	53.6 (52.2–54.9)	56.0 (54.1–58.0)	60.5 (58.8–62.2)	*P* = 0.097	**P** < 0.001^*^	**P** = 0.002^*^
Cognitive function (95% CI)	63.7 (62.1–65.4)	67.3 (65.1–69.6)	70.0 (68.0–72.0)	**P** = 0.030^*^	**P** < 0.001^*^	*P* = 0.190
Health distress (95% CI)	58.2 (56.5–59.9)	59.0 (56.6–61.3)	64.6 (62.7–66.5)	*P* = 0.855	**P** < 0.001^*^	**P** = 0.001^*^

^*^Denotes significant *P* value of <0.05.

**Table 3:** Predicting the PHC, MHC, and depression with meditation subgroups.

Dependent	Covariates	*B*	95% CI		*P*	Adjusted *R* ^2^
			Lower	Upper		
MHC	Age, years	0.10	0.02	0.19	0.022	0.030
	Gender, male	0.62	−1.78	3.02	0.613	
	Secondary school or less^*^	−7.31	−9.94	−4.67	<0.001	
	Vocational training^*^	−4.45	−7.42	−1.49	0.003	
	Bachelor degree^*^	−1.64	−4.03	0.75	0.179	
	Meditates once or more per week^†^	4.80	2.65	6.94	<0.001	
	Meditates less than once per week^†^	0.78	−1.55	3.11	0.513	

PHC	Age, years	0.42	0.33	0.51	<0.001	0.081
	Gender, male	2.67	0.22	5.11	<0.001	
	Secondary school or less^*^	−8.60	−11.38	−5.82	<0.001	
	Vocational training^*^	−7.31	−10.37	−4.24	<0.001	
	Bachelor degree^*^	−2.16	−2.71	2.28	0.87	
	Meditates once or more per week^†^	2.99	0.75	5.24	0.009	
	Meditates less than once per week^†^	2.69	0.24	5.15	0.031	

		OR	Lower	Upper	*P*	

Depression	Age, years	0.99	0.98	1.00	0.092	
	Gender, male	1.01	0.75	1.34	0.976	
	Secondary school or less^#^	2.07	1.49	2.88	<0.001	
	Vocational training^#^	1.88	1.31	2.70	<0.001	
	Bachelor degree^#^	1.27	0.92	1.75	0.146	
	Once or more per week^‡^	0.52	0.39	0.69	<0.001	
	Less than once per week^‡^	0.88	0.67	1.16	0.366	

Disability	Age, years	1.08	1.07	1.09	<0.001
	Gender, male	1.04	0.83	1.31	0.726	
	Secondary school or less^#^	1.57	1.22	2.02	<0.001	
	Vocational training^#^	1.72	1.30	2.27	<0.001	
	Bachelor degree^#^	1.04	0.82	1.31	0.773	
	Once or more per week^‡^	1.18	0.96	1.44	0.108	
	Less than once per week^‡^	0.75	0.59	0.94	0.014	

*B* is the unstandardized regression coefficient and 95% CI is the 95% confidence interval of the unstandardized regression coefficient.

^*^Compared to postgraduate qualification.

^†^Compared to “never meditates.”

OR is the odds ratio and 95% CI is the 95% confidence interval of the odds ratio.

^#^Compared to postgraduate qualification.

^‡^Compared to “never meditates.”
